# The DICA endoscopic score and the CODA clinical score may predict the severity of acute diverticulitis and the risk of hospitalisation: results from an international multicentre prospective cohort study

**DOI:** 10.1007/s10151-026-03324-6

**Published:** 2026-05-05

**Authors:** Antonio Tursi, Daniele Piovani, Giovanni Brandimarte, Francesco Di Mario, Gisella Figlioli, Dan L. Dumitrascu, Enio Chaves Oliveira, Valerio Papa, Savvas Papagrigoriadis, Ieva Stundiene, Matthias Christian Reichert, Jaroslaw Regula, Walter Elisei, Marcello Picchio, Gabrio Bassotti, Mauro Bafutto, Giovanni Latella, Giovanni Maconi, Stefanos Bonovas, Alfredo Papa, Silvio Danese

**Affiliations:** 1 Territorial Gastroenterology Service, ASL BAT, Andria, BT Italy; 2 Department of Medical and Surgical Sciences, School of Medicine, Catholic University, Rome, Italy; 3 Department of Biomedical Sciences, Humanitas University, Milan, Italy; 4 IRCCS Humanitas Research Hospital, Rozzano, Milan Italy; 5 Humanitas University Consortium, Rome, Italy; 6 Department of Medical and Surgical Sciences, Gastroenterology Unit, University of Parma, Parma, Italy; 7 2nd Medical Department, Iuliu Hatieganu” University of Medicine and Pharmacy, Cluj-Napoca-Napoca, Romania; 8 Department of Colorectal Surgery, Federal University of Goiás, Goiânia, Goiás Brazil; 9 Department of Medical and Surgical Sciences and Translational Medicine, “Sapienza” University, St. Andrea Hospital, Rome, Italy; 10 Department of Colorectal Surgery, King’s College Hospital, London, UK; 11 Institute of Clinical Medicine, Vilnius University Hospital, Vilnius, Lithuania; 12 Department of Medicine II, Saarland University Medical Center, Homburg, Germany; 13 Department of Gastroenterology, Hepatology and Clinical Oncology, Centre of Postgraduate Medical Education, Maria Sklodowska-Curie National Research Institute of Oncology, Warsaw, Poland; 14 Division of Gastroenterology, “S. Camillo” Hospital, Rome, Italy; 15 Division of Surgery, “P. Colombo” Hospital, ASL RM6, Velletri (Rome), Italy; 16 Department of Medicine and Surgery, Gastroenterology and Hepatology Unit, Santa Maria Della Misericordia” University Hospital, University of Perugia, Perugia, Italy; 17 Institute of Gastroenterology and Digestive Endoscopy, Goiânia, Goiás Brazil; 18 Division of Gastroenterology, Hepatology and Nutrition, Department of Life, Health & Environmental Sciences, San Salvatore Hospital, University of L’Aquila, L’Aquila, Italy; 19 Division of Gastroenterology, “L. Sacco” University Hospital, Milan, Italy; 20 Department of Internal Medicine and Gastroenterology, Fondazione Policlinico A. Gemelli, IRCCS, Rome, Italy; 21 Gastroenterology and Endoscopy, IRCCS Ospedale “San Raffaele” and University “Vita-Salute San Raffaele”, Milan, Italy; 22 Digestive Diseases Centre (CEMAD), Department of Medical and Surgical Sciences, Policlinico Universitario “A. Gemelli” Foundation, IRCCS, Largo Francesco Vito, 1, 00168 Rome, Italy

**Keywords:** Acute diverticulitis, CODA score, DICA classification, Hospital stay

## Abstract

**Background:**

We assessed whether baseline endoscopic and clinical scores of diverticular disease (DD) may predict the severity of acute diverticulitis (AD), hospital admission and length of hospital stay (HS).

**Methods:**

We conducted a 3-year, multicentre, prospective cohort study involving 2215 patients. DD was scored according to the Diverticular Inflammation and Complication Assessment (DICA) classification and the Combined Overview on Diverticular Assessment (CODA) score.

**Results:**

Higher baseline DICA and CODA scores were associated with increased risk of complicated AD [relative risk ratio (RRR) = 5.57; 95% confidence interval (CI): 3.42–9.09 and RRR = 5.17; 95% CI: 3.01–8.88, respectively]. An average HS of 4.62 days (95% CI: 2.93–6.27) for DICA 1, 5.57 days (95% CI: 4.29–6.85) for DICA 2 and 6.73 days (95% CI: 5.81–7.64) for DICA 3 was recorded; an average HS of 4.14 days (95% CI: 2.32–5.96) for CODA A, 5.12 days (95% CI: 3.67–6.56) for CODA B and 6.32 days (95% CI: 5.35–7.30) for CODA C was recorded.

**Conclusions:**

DICA and CODA scores may predict the severity of AD and the length of the HS.

**Supplementary Information:**

The online version contains supplementary material available at 10.1007/s10151-026-03324-6.

## Introduction

Diverticular disease (DD) of the colon is among the most frequent gastro-intestinal (GI) diagnoses worldwide [1]. Its incidence and prevalence are increasing worldwide [2], with substantial direct and indirect costs [3]. For example, in the USA, the annual incidence is approximately 180 per 100,000 people, resulting in almost 200,000 hospital admissions annually (ranking sixth among hospital admissions for digestive diseases) and an estimated health care expenditure of more than 6.3 billion dollars/year [4]. Overall, the in-hospital management of these patients is increasing worldwide [5–8], and the in-hospital mortality rate approaches 7% [8].

Colonic diverticula are well known as the most common anatomical alteration detected during colonoscopy [1]. Notwithstanding, the first endoscopic classification of diverticulosis/diverticular disease, called Diverticular Inflammation and Complication Assessment (DICA), was developed in 2015 [9]. This classification has followed a strict process of validation [10, 11], in which it has been improved by incorporating clinical (age, severity of abdominal pain at diagnosis, and type and severity of bowel habits) [12, 13] and laboratory (faecal calprotectin) [14] parameters. The final results showed that, when combined with clinical and laboratory parameters, the DICA score can predict disease evolution, including the risk of acute diverticulitis (AD) and the need for surgery.

However, whether the DICA score, and its clinical evolution named Combined Overview on Diverticular Assessment (CODA) [12], may predict the severity of AD, the risk of hospitalisation and the length of the hospital stay is still not known. Indeed, only a retrospective study has found that the DICA score may predict the severity of AD [15], with no prospective data currently available.

We therefore aimed to prospectively assess whether the DICA and CODA scores could predict the severity of AD, the risk of hospitalisation for AD and the length of hospital stay.

## Materials and methods

### Study design and population

This study is a post hoc analysis of a multicenter, prospective, international cohort study conducted in 43 gastroenterology and endoscopy centres located in Europe and South America, previously described elsewhere [12]. In brief, the cohort consists of 2215 patients with newly diagnosed colonic diverticula. Patients were followed for up to 3 years or until follow-up was interrupted. All participants underwent baseline endoscopic evaluation, and the presence of diverticula was classified using the DICA and CODA scores. The DICA classification assigns a numerical score based on four main endoscopic criteria and additional sub-items. The total score stratifies patients into three categories: DICA 1 (≤ 3 points), DICA 2 (4–7 points) and DICA 3 (> 7 points) [12]. Also, the CODA score assigns a numerical score to three main categories, namely age at diagnosis, DICA score and severity of the abdominal pain measured on a ten-point visual analogue scale (VAS), reflecting symptom severity at the time of diagnosis. The resulting total score stratifies patients into three categories: CODA A (3–9 points), CODA B (10–16 points) and CODA C (> 16 points) [12]. Further details on the study design, eligibility criteria and the DICA and CODA classification can be found in the online supplemental appendix and Supplementary Tables S1 and S2. The aims of this analysis were:

(1) To assess whether the severity of DICA and CODA scores at baseline may predict the severity of AD that occurred during the follow-up, in terms of uncomplicated versus complicated AD. The AD diagnosis was made according to abdominal computerised tomography scan: uncomplicated AD was defined as inflammation of the colonic wall harbouring diverticula with fat stranding, and without complications such as abscesses, stenosis or fistulas; complicated AD was defined as inflammation of the colonic wall harbouring diverticula with fat stranding, and with complications such as abscesses, stenosis or fistulas [12];

(2) To assess whether the severity of the DICA and CODA scores at baseline may predict the length of hospital stay for AD.

### Statistical analysis

Descriptive statistics were summarised using median and interquartile range (IQR) for continuous variables and count and percentage for categorical variables.

Association between baseline severity and AD risk: multinomial logistic regression models were used to assess the association between baseline diverticular disease severity (DICA and CODA scores) and the risk and severity of AD, categorised as a three-level outcome (no AD, uncomplicated AD, complicated AD). To retain simplicity in interpretation while capturing a linear trend across increasing severity levels, DICA and CODA scores were modelled in separate regressions as three-level continuous exposures, assuming proportionality of predicted risk across levels [12]. The model, including the DICA score as the primary exposure, was adjusted for age, sex and body mass index (BMI). As the CODA score incorporates age, its model was adjusted only for sex and BMI. These covariates were selected a priori on the basis of their clinical relevance and potential confounding role in the relationship between baseline disease severity and subsequent AD risk. The number of these covariates was deliberately restricted given the limited number of events to ensure models stability and avoid overfitting. Results are reported as relative risk ratios (RRRs), interpreted as the relative change in the probability of developing uncomplicated or complicated AD for each one-unit increase in DICA or CODA score, respectively.

Association between baseline severity and hospital stay: ordinary least-squares regression models were used to evaluate the association between baseline DICA and CODA scores and the length of hospital stay (in days) among hospitalised AD patients. Owing to severe right-skewness, the length of hospital stay was log-transformed to improve residual normality. Separate models were fitted for DICA and CODA scores, each treated as a continuous variable. The DICA model was adjusted for age, sex and the Charlson comorbidity index. The model for CODA was adjusted for sex and the Charlson comorbidity index, as age is a component of the CODA score. Adjusted predictions for the length of stay were derived using marginal estimates. For each value of the DICA or CODA score, the model computed the expected mean number of days, holding the other covariates at their observed distributions. Because the models were fitted on the log scale, predicted values were back-transformed via exponentiation to obtain clinically interpretable adjusted average hospitalisation durations on the original scale, along with 95% CIs. These estimates represent the expected average hospitalisation duration for different levels of baseline DICA or CODA scores. To assess whether the association between DICA or CODA scores and length of stay differed by AD severity at admission (uncomplicated versus complicated), an interaction term between the continuous score and AD subtype was included in the respective models. Effect modification was evaluated using the *p*-value for the interaction term.

All statistical tests were two-sided, and *p*-value < 0.05 was considered statistically significant. Analyses were conducted using Stata version 19 (StataCorp, College Station, TX).

## Results

Of the 2215 patients initially enrolled, only 13 (0.6%) were excluded owing to missing outcome data (DICA 1, *n* = 8; DICA 2, *n* = 5; DICA 3, *n* = 0; CODA A, *n* = 5; CODA B, *n* = 6; CODA C, *n* = 2). These patients were not concentrated in higher DICA and CODA severity categories, minimising the risk of attrition bias. Consequently, 2202 patients with available information on AD status (no AD, uncomplicated AD or complicated AD) were included in the analyses. Baseline demographic and clinical characteristics of the included patients are reported in Table 1.

**Table 1:** Baseline demographic and clinical characteristics of the study population (*N* = 2202)

Baseline characteristic	Median (IQR) or *N* (%)
Age, years	66 (58−74)
≥ 65	1,226 (55.7)
Sex, female	1,140 (51.8)
BMI, kg/m^2^	26 (24−29)
≥ 30	454 (20.6)
Smoking	
Smokers	512 (23.2)
Non-smokers	1,355 (61.5)
Former smokers	335 (15.2)
Appendectomy	535 (24.4)
Presence of co-morbidities	
Charlson’s score	3 (2−4)
Charlson’s score > 3	679 (30.8)
Presence of any symptom	1,683 (76.4)
Cumulative symptom score^1^	7 (1−12)
> 7	1,014 (46.0)
Abdominal pain score	2 (0−4)
> 2	907 (41.2)
Meteorism score	2 (0−4)
> 2	991 (45.0)
Constipation score	0 (0−3)
> 2	601 (27.3)
Diarrhoea score	0 (0−2)
> 2	411 (18.7)
Surgery	43 (2.0)
DICA score	
1	1372 (62.3)
2	593 (26.9)
3	237 (10.8)
CODA score	
A	1149 (52.2)
B	628 (28.5)
C	425 (19.3)
Faecal calprotectin^2^, μg/g	20 (9.3−60)

*N*, number; IQR, interquartile range; BMI, body mass index; DICA, Diverticular Inflammation and Complication Assessment; CODA, Combined Overview on Diverticular Assessment

Values are expressed as median (interquartile range) for continuous variables and as number (percentage) for categorical variables

^1^The cumulative symptom score ranges from 0 to 40 points. It is obtained by adding the points regarding abdominal pain, meteorism, constipation and diarrhoea as measured on a ten-point visual analogue scale

^2^Faecal calprotectin was available for 910 patients

Patients were predominantly females (51.8%) with a median age of 66 years (IQR, 58‒74 years). They were slightly overweight (median BMI, 26 kg/m^2^; IQR, 24‒29 kg/m^2^), and 23.2% of them were smokers. Most patients (62.3%) were classified as DICA 1, 26.9% as DICA 2 and 10.8% as DICA 3. On the basis of the DICA score, age and abdominal pain score, the CODA score was calculated for each patient (median CODA score, 8; IQR, 6‒14). According to CODA categories, 52.2% of the patients were classified as CODA A, 28.5% as CODA B and 19.3% as CODA C.

During a median follow-up of 3 years, 162 patients developed AD (7.3%). Of them, 129 patients (79.6%) developed uncomplicated AD and 33 (20.4%) complicated AD. Among patients with uncomplicated AD, 78 (60.46%) were not hospitalised, 46 (35.66%) were hospitalised for uncomplicated AD and 5 (3.88%) were hospitalised for other reasons.

All patients with a diagnosis of complicated AD were hospitalised. Higher baseline DICA and CODA scores were associated with an increased risk and severity of AD (Table 2). Each one-unit increase in the DICA score was associated with a higher risk of both uncomplicated AD (RRR = 2.54; 95% CI: 2.01–3.21) and complicated AD (RRR = 5.57; 95% CI: 3.42–9.09) compared with no AD. When directly comparing complicated with uncomplicated AD, a higher baseline DICA score remained significantly associated with more severe outcomes (RRR = 2.20; 95% CI: 1.29–3.73). Similarly, each one-level increase in CODA score was associated with a significantly higher risk of developing uncomplicated AD (RRR = 2.52; 95% CI: 2.01–3.16) and complicated AD (RRR = 5.17; 95% CI: 3.01–8.88) compared with no AD. When comparing complicated with uncomplicated AD, higher baseline CODA scores remained associated with greater AD severity (RRR = 2.05; 95% CI: 1.15–3.66).

**Table 2:** Associations between baseline DICA and CODA scores and the risk and severity of acute diverticulitis

Score	Outcome	RRR	(95% CI)	*p*
DICA				
	Uncomplicated AD versus no AD	2.54	(2.01−3.21)	< 0.001
	Complicated AD versus no AD	5.57	(3.42−9.09)	< 0.001
	Complicated versus uncomplicated AD	2.20	(1.29−3.73)	0.004
CODA				
	Uncomplicated AD versus no AD	2.52	(2.01−3.16)	< 0.001
	Complicated AD versus no AD	5.17	(3.01−8.88)	< 0.001
	Complicated versus uncomplicated AD	2.05	(1.15−3.66)	0.015

RRR, relative risk ratio; CI, confidence interval; AD, acute diverticulitis

Relative risk ratios were estimated using multivariable multinomial logistic regression. DICA and CODA scores were modelled as continuous exposures. Models with the DICA score as the primary exposure were adjusted for age, sex and BMI; models with the CODA score were adjusted for sex and BMI. The reported RRRs represent the relative change in the probability of developing uncomplicated or complicated AD for each one-unit increase in the DICA or CODA scores, respectively

Among the 162 patients who developed AD during follow-up, 84 (51.8%) required hospitalisation, and length of stay data were available for 81 of these individuals. Higher baseline disease severity was associated with a longer hospitalisation. After adjustment by age, sex and Charlson comorbidity index, each one-unit increase in the baseline DICA score was associated with a statistically significant increase in length of hospital stay (*β* = 0.19; standard error (SE) = 0.07; *P* = 0.012). Back-transformed adjusted predictions corresponded to an average hospital stay of 4.62 days (95% CI: 2.93–6.27) for DICA 1, 5.57 days (95% CI: 4.29–6.85) for DICA 2 and 6.73 days (95% CI: 5.81–7.64) for DICA 3. The observed association did not differ between patients hospitalised for uncomplicated and those hospitalised for complicated AD (*P*_interaction_ = 0.52). Similarly, after adjustment for sex and the Charlson comorbidity index, each one-unit increase in baseline CODA score was associated with a more extended hospital stay (*β* = 0.21; SE = 0.08; *P* = 0.016). Predicted average hospital stays increased from 4.14 days (95% CI: 2.32–5.96) for CODA A, 5.12 days (95% CI: 3.67–6.56) for CODA B and 6.32 days (95% CI: 5.35–7.30) for CODA C. There was no evidence that this association differed according to whether the AD was uncomplicated or complicated (*P*_interaction_ = 0.70).

## Discussion

The incidence and prevalence of AD are increasing worldwide [2], with high costs [3]. The trend already reported in the USA [4] has been confirmed in other countries as well. For example, an Italian nationwide study showed a significant increase in the number and the rate of hospital admissions for AD between 2008 and 2015 (about 30% overall and 3.2% per year) [6]; in the same period, the global number of hospital admissions in Italy significantly declined by 25%. These temporal trends suggest actual changes in the epidemiology of AD, including changes in disease incidence and severity, rather than a lower threshold for hospitalisation. Predictive factors are therefore needed to assess disease severity, the risk of hospitalisation and the length of hospital stay in patients with AD. Some parameters have been investigated in the past to identify which factors may influence the severity of AD: risk of complications, organ dysfunction, peritonitis and abscess size greater than 6 cm, as well as higher Clinical Frailty Score (CFS) and sepsis, were identified as independent predictors of poor outcome [16, 17]. Significantly, age did not appear to be a predictor of poor outcome [17].

This prospective, international study investigated other factors that had not yet been assessed. We evaluated the severity of the endoscopic appearance in patients at the time of first diagnosis of diverticulosis/DD, because in most cases, the diagnosis is currently made by endoscopy. We investigated whether the baseline severity of the DICA endoscopic score and its clinical evolution, as measured by the CODA score, may be predictive of the above-mentioned outcomes. We chose to investigate these parameters because both were found to be able to predict the risk of AD and surgery in patients with diverticulosis at their first diagnosis [12]. This study found that both DICA and CODA scores are predictive of AD severity and length of hospital stay. This means that, if we score a patient at the first diagnosis of diverticulosis using the DICA and CODA scores, we can predict not only the risk of AD and surgery but also the severity of AD and the length of hospital stay in case of hospitalisation. Also, adjusting for sex and the Charlson comorbidity index (age is adjusted only for DICA score, because age is a component of the CODA score), these two parameters remain strong predictors of AD severity and length of hospital stay, confirming their significant role in predicting the outcome of this disease.

Another interesting finding of this study is that, among patients with uncomplicated AD, most were not hospitalised. It is now well established that uncomplicated AD can be managed on an outpatient basis in most cases, without negatively impacting disease outcomes (disease recurrence, hospitalisation and risk of surgery) [18]. Furthermore, this approach also helps save costs in patient management, as demonstrated by an analysis conducted in Sweden [19]. This prospective analysis confirmed this approach: most patients with uncomplicated AD can be managed as outpatients, and inpatient management should be reserved for patients with significant risk factors (sepsis, significant comorbidities and immunosuppressant use) [1].

Of course, this study has some limitations. The first is that the limited number of events reduced the statistical power, particularly for complicated AD. To maintain an acceptable events-per-variable ratio and avoid overfitting, we deliberately restricted the number of covariates in the multinomial regression models. Consequently, a reduction in statistical power, limited precision of the estimates and potential residual confounding cannot be excluded. Therefore, our results should be interpreted cautiously and considered to be hypothesis-generating, especially for complicated AD. Second, information about treatment for AD and the outcome after hospital discharge was not available. Unfortunately, this is likely the study’s main limitation. In fact, the original protocol did not include the analysis of treatment for patients after the occurrence of AD, and therefore, it cannot be further investigated. At the same time, another limit is that we do not know how the uncomplicated AD patients were managed at home. In other words, we do not know whether these patients were treated with oral antibiotics or only with support therapy. This is because it is now well established that antibiotic therapy in outpatient management of uncomplicated AD does not seem to give significant advantages over the support therapy [20]. Finally, while our analyses indicate strong prognostic associations, we did not assess formal measures of predictive performance, such as discrimination (e.g. area under the curve) and calibration metrics, which would require dedicated external validation studies.

Despite these limitations, this study found that DD severity at diagnosis, as measured by DICA and CODA scores, can predict AD severity and length of hospital stay when necessary. New, well-designed studies should address potential residual confounding, data gaps, the limited number of events and the need for formal validation of predictive performance.

## Supplementary Information

Below is the link to the electronic supplementary material.

Supplementary file1 (DOCX 16 KB)

Supplementary file2 (DOC 73 KB)

Supplementary file3 (DOC 38 KB)

## Data Availability

Data are available upon reasonable request.
