# Supplementary material for: The DICA endoscopic score and the CODA clinical score may predict the severity of acute diverticulitis and the risk of hospitalisation: results from an international multicentre prospective cohort study
Source: Tech Coloproctol. 2026 May 5;30(1):85. doi: 10.1007/s10151-026-03324-6 (PMC13319915; doi:10.1007/s10151-026-03324-6)
Supplement: Supplementary file 1 — Supplementary file1 (DOCX 16 KB) [file 10151_2026_3324_MOESM1_ESM.docx]

**SUPPLEMENTARY METHODS**

***Inclusion and exclusion criteria***

Inclusion criteria were:

age >18 years;

first endoscopic diagnosis of diverticulosis

Exclusion criteria were:

radiological signs (by abdominal CT or by ultrasounds) of acute diverticulitis (defined as inflammation of colonic wall harboring diverticula with fat stranding, and with or without complications such as abscesses, stenosis or fistulas, namely uncomplicated or complicated diverticulitis);

inflammatory bowel diseases;

ischemic colitis;

prior colonic resection;

patients with severe liver failure (Child-Pugh C);

patients with severe kidney failure;

pregnant women;

women of childbearing potential not using a highly effective method of contraception;

patients with current use or who have received any laxative agents <2 weeks prior to the enrollment;

patients with recent use or who have received mesalamine compounds <2 weeks prior to the enrollment;

patients with current use or who have received any probiotic agents 2 weeks prior to the enrollment; nonsteroidal anti-inflammatory drug (NSAID) use (except for acetyl-salycilic acid ≤100 mg/day) <1 week prior to the enrollment;

patients who have received treatment with antibiotics (even those not absorbed) <2 weeks prior to the enrollment;

inability to comply with study protocol and to give informed consent to the procedure;

patients with or history of cancer, of any origin, within 5 years before enrollment;

history of alcohol, drug, or chemical abuse; any severe patological condition interfering with the proper study execution.

Further data about the study's design were reported in the original study (1).

***Ethic statement***

The study has been conducted according to the [World Medical Association Declaration of Helsinki](http://www.wma.net/en/30publications/10policies/b3/index.html) of the 1975. It was approved by the Ethic Committee of the coordinator centre (protocol number 87/CE/2015) and of all participating centres. All study participants provided informed written consent prior to endoscopic investigation and to take part in this study. The original study was recorded at www.ClinicalTrials.gov (NCT02758860).

***Reference***

1) Tursi A, Brandimarte G, Di Mario F et al.; DICA International Group. [Prognostic performance of the 'DICA' endoscopic classification and the 'CODA' score in predicting clinical outcomes of diverticular disease: an international, multicentre, prospective cohort study.](https://pubmed.ncbi.nlm.nih.gov/34702716/) Gut 2022;71: 1350-1358
