# Supplementary material for: The DICA endoscopic score and the CODA clinical score may predict the severity of acute diverticulitis and the risk of hospitalisation: results from an international multicentre prospective cohort study
Source: Tech Coloproctol. 2026 May 5;30(1):85. doi: 10.1007/s10151-026-03324-6 (PMC13319915; doi:10.1007/s10151-026-03324-6)
Supplement: Supplementary file 2 — Supplementary file2 (DOC 73 KB) [file 10151_2026_3324_MOESM2_ESM.doc]

**Supplementary Table 1.** Items assessed in constructing DICA (Diverticular Inflammation and Combination Assessment) endoscopic classification.

**Right** (1 point)

**Left** (2 points)

**Location**

**Number of diverticula**

**(in each district)**

**Inflammation**

**Complications**

**Grade I**: ≤ 15 diverticula (0 points)

**a**: absence of signs of inflammation

(o points)

**R**: rigidity (4 points)

**Grade II**: >15 diverticula (1 point)

**b**: edema/hyperemia (1 point)

**ST**: stenosis (4 points)

**c**: erosions (2 points)

**P**: pus (4 points)

**d:** SCAD (Segmental Colitis Associated with Diverticulosis) (3 points)

**B**: bleeding (4 points)

| **DICA SCORE** | **Numeric values** |
| --- | --- |
| **DICA1** | 1-3 points |
| **DICA 2** | 4-7 points |
| **DICA 3** | >7 points |
