# Supplementary material for: The DICA endoscopic score and the CODA clinical score may predict the severity of acute diverticulitis and the risk of hospitalisation: results from an international multicentre prospective cohort study
Source: Tech Coloproctol. 2026 May 5;30(1):85. doi: 10.1007/s10151-026-03324-6 (PMC13319915; doi:10.1007/s10151-026-03324-6)
Supplement: Supplementary file 3 — Supplementary file3 (DOC 38 KB) [file 10151_2026_3324_MOESM3_ESM.doc]

**Supplementary Table 2.** Items assessed in constructing CODA (Combined Overview on Diverticular Assessment) score.

| Endoscopic score | Abdominal pain score | Patient age |
| --- | --- | --- |
| **DICA 1** (7 points) | **1** (1 point) | **<65 years** (0 points) |
| **DICA 2** (14 points) | **2** (2 points) | **≥65 years** (−4 points) |
| **DICA 3** (21 points) | **3** (3 points) |  |
|  | **4** (4 points) |  |
|  | **5** (5 points) |  |
|  | **6** (6 points) |  |
|  | **7** (7 points) |  |
|  | **8** (8 points) |  |
|  | **9** (9 points) |  |
|  | **10** (10 points) |  |

| **CODA SCORE** | **Numeric values** |
| --- | --- |
| **CODA A** | 3−9 points |
| **CODA B** | 10−16 points |
| **CODA C** | >16 points |

*Footnotes*: Abdominal pain as measured on a 10-point visual analogue scale.

*Abbreviations:* DICA, Diverticular Inflammation and Complication Assessment
